# Controlled Synthesis of Pd/Pt Core Shell Nanoparticles Using Area-selective Atomic Layer Deposition

**DOI:** 10.1038/srep08470

**Published:** 2015-02-16

**Authors:** Kun Cao, Qianqian Zhu, Bin Shan, Rong Chen

**Affiliations:** 1 State Key Laboratory of Digital Manufacturing Equipment and Technology, School of Mechanical Science and Engineering; 2 State Key Laboratory of Material Processing and Die and Mould Technology, School of Materials Science and Engineering, Huazhong University of Science and Technology, 1037 Luoyu Road, Wuhan, Hubei, PR China 430074

## Abstract

We report an atomic scale controllable synthesis of Pd/Pt core shell nanoparticles (NPs) via area-selective atomic layer deposition (ALD) on a modified surface. The method involves utilizing octadecyltrichlorosilane (ODTS) self-assembled monolayers (SAMs) to modify the surface. Take the usage of pinholes on SAMs as active sites for the initial core nucleation, and subsequent selective deposition of the second metal as the shell layer. Since new nucleation sites can be effectively blocked by surface ODTS SAMs in the second deposition stage, we demonstrate the successful growth of Pd/Pt and Pt/Pd NPs with uniform core shell structures and narrow size distribution. The size, shell thickness and composition of the NPs can be controlled precisely by varying the ALD cycles. Such core shell structures can be realized by using regular ALD recipes without special adjustment. This SAMs assisted area-selective ALD method of core shell structure fabrication greatly expands the applicability of ALD in fabricating novel structures and can be readily applied to the growth of NPs with other compositions.

Bimetallic nanoparticles (NPs) have attracted great attention due to their unique properties for catalytic applications^1^,^2^,^3^,^4^,^5^,^6^,^7^,^8^. It is found that the formation of core shell NPs could further enhance the activity, selectivity and stability^9^,^10^,^11^. Compared with the physical mixture of monometallic NPs or the alloyed bimetallic NPs, the enhanced properties of core shell structure may originate from the lattice strain, bonding interaction and electron transfer due to the unique core shell interface^12^,^13^. For instance, the Pd/Pt core shell NPs show enhanced activity for the oxygen reduction reaction and methanol oxidation^8^,^12^, whose enhancement of catalytic performance can be attributed to the lowering of the surface electronic d-band center^14^,^15^,^16^. From cost perspective, replacing the noble metal cores with a less expensive metal could also lower the overall materials cost and make commercial applications of core shell NPs more favorable.

Synthesizing core shell nanoparticles (NPs) with well controlled shell thickness and composition is of great importance in optimizing their reactivity^17^,^18^,^19^,^20^,^21^. Wet chemical synthesis methods have been tried to fabricate the bimetallic core shell NPs by commonly reducing a second metal onto the preformed cores^2^,^9^. However, the size of as synthesized core shell NPs is generally larger than 10 nm^22^, which is not ideal for some chemical reactions that require a larger surface area. In addition, it still remains a challenge to fabricate core shell NPs with precisely controlled composition and shell thickness due to the difficulty of purification and separation for the solvent based synthesis method. Recently, gas phase methods such as atomic layer deposition (ALD) have been applied to the synthesis of supported monometallic NPs such as Pd, Pt and so on^23^,^24^,^25^,^26^,^27^. The ALD method also allows well dispersed NPs directly grown in porous structures as well as on high aspect ratio substrate^28^,^29^. NPs grown by ALD show well controlled size by tuning the ALD process^30^,^31^,^32^,^33^. However, reports on the fabrication of core shell NPs through ALD are very limited and often involves relatively complex processes. Kessels *et al.* demonstrated the selective growth of Pt on Pd cores by ALD method. To achieve the core shell structure, a low oxygen pressure of 7.5 mTorr which is about two orders of magnitude lower than regular Pt ALD process must be used to avoid the formation of monometallic NPs^34^. Elam *et al.* also reported that core shell NPs could be obtained by carefully adjusting the ALD temperature and the proper choice of suitable counter reactant^35^,^36^. In both cases, the core shell NPs growth requires either special tuning of ALD parameters or development of new ALD recipes that takes much effort and time. It is also possible that the modified ALD pressure or temperature required for the core shell growth might not be compatible with existing experimentally accessible conditions or may lead to undesirable side reactions. Thus it is much desirable to develop a robust and universal ALD process for the core shell NP growth.

We report here the successful synthesis of core shell NPs using an area-selective ALD (AS-ALD) technique. Previous studies reported that ODTS SAMs was stable under vacuum condition up to 300°C. The closely packed ODTS can be used as a resist in AS-ALD process to block deposition of HfO_2_, ZrO_2_, Pt. etc.^37^,^38^,^39^ Bent *et al.* also reported that the pinhole structures on unsaturated ODTS SAMs could influence monometallic Pt NPs nucleation behavior such as density and size distribution^40^. In this study we utilize similar approach to form pinhole structures, and grow core shell NPs using regular ALD recipes without special adjustments. This new AS-ALD synthesis strategy utilizes pinholes on the unsaturated ODTS SAMs layer as active sites for the initial ALD nucleation while the closely packed ODTS layer blocks the precursor deposition during the cores formation process. ODTS SAMs also enables selective growth of the second metal onto the cores, forming uniformly distributed core shell NPs. The size, shell thickness and composition of the NPs can be controlled precisely by varying the ALD cycles and significantly expands the applicability of this method. We demonstrate the realization of AS-ALD NP growth process by fabricating Pd/Pt and Pt/Pd core shell structures, which can be readily extended to the growth of other core shell structures.

## Results and Discussion

**Figure 1:**
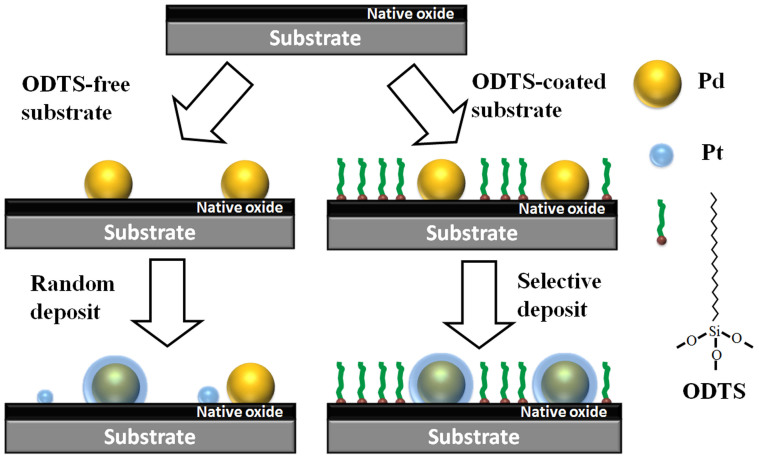
Schematic illustration for fabricating the core shell nanoparticles through AS-ALD on ODTS modified substrate.

Figure 1 is a schematic illustration of the synthesis strategy of the Pd core/Pt shell NPs exploiting AS-ALD growth on the ODTS SAMs modified substrate. First an ODTS SAMs were formed on the native oxide terminated substrate, the SAMs formation time was purposely controlled to be less than the saturation time to obtain pinholes. These pinholes were ODTS uncovered sites and exposed the reactive hydroxyl (−OH) functional group of the silicon substrate^41^. Other parts were covered with closely packed ODTS molecules which have inert methyl (−CH_3_) end groups^42^,^43^. During the first stage of core formation, the pinholes with active hydroxyl groups could initiate the nucleation of Pd to form the metal cores. During the second ALD stage for shell formation, the Pt precursors could only chemically bind to Pd cores deposited in the first stage, but not to the closely packed SAMs layer. As a result, Pt was selectively deposited onto the Pd nuclei and formed Pd/Pt core shell NPs. In comparison, the nucleation of Pt on the substrates could hardly be avoided on a bare silicon substrate without introducing special adjustment of ALD reaction conditions, resulting in a mixed phase formation of Pt, Pd and Pd/Pt NPs.

**Figure 2:**
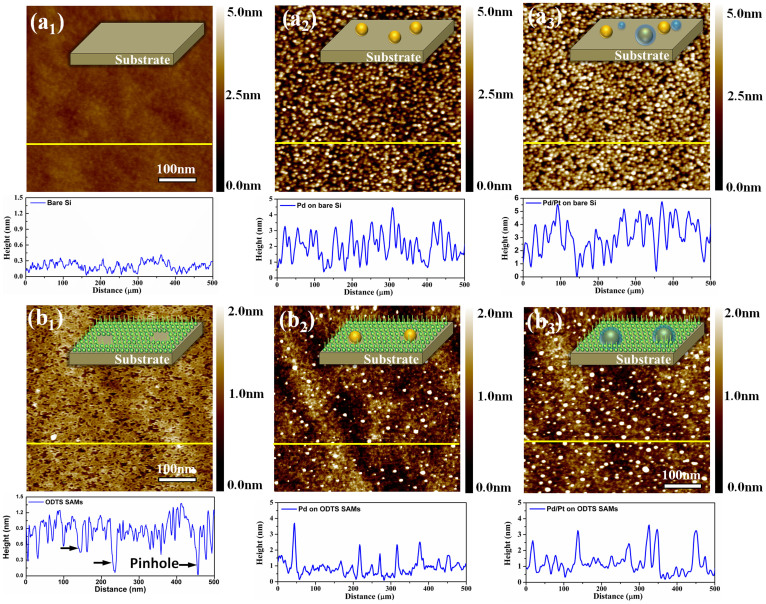
AFM images of (a_1_) ODTS-free Si substrate, (a_2_) 300 cycles of Pd on ODTS-free Si substrate, (a_3_) 300 cycles Pd + 25 cycles Pt on ODTS-free Si substrate, (b_1_) 2 h ODTS-coated Si substrate, (b_2_) 300 cycles of Pd on 2 h ODTS-coated Si substrate, (b_3_) 300 cycles Pd + 25 cycles Pt on 2 h ODTS-coated Si substrate. Below the images show the sectional analysis of the corresponding samples.

Atomic force microscope (AFM) was used to reveal the correlation between the NPs density (defined as NPs' counts per unit area) and the number of pinholes in ODTS film. The results are shown in Figure 2. The surface morphology of ODTS-free Si substrate (Figure 2a1) is flat with ~0.3 nm surface roughness. The ODTS-coated substrate (2 h dip time for SAMs growth, Figure 2b1) is less smooth with significant number of nanometer scale pinholes. Those pinholes are uncovered sites that could enable the precursors to penetrate through the organic film and react with surface hydroxyl groups^41^. Figure 2a2, b2 represent samples with 300 cycles of Pd grown on ODTS-free and ODTS-coated substrates, respectively. On ODTS-free substrate, the deposited NPs density is 5.1 × 10^3^/um^2^, while the NPs density reduces to 2.4 × 10^3^/um^2^ when the same ALD process is conducted on ODTS modified substrate. The pinholes density of ODTS-coated substrate extracted from figure 2b1 was ~2.9 × 10^3^/um^2^ which is about the same with the density of Pd NPs on ODTS-coated substrate in Figure 2b2. This illustrates that the pinholes on the unsaturated ODTS layer act as nucleation sites, while the areas covered with closely packed ODTS molecular effectively block precursor chemisorption and limit the nucleation of metal NPs. Figure 2a3, b3 represent the corresponding samples in Figure 2a2, b2 after 25 cycles Pt ALD process. From the cross section height profile from Figure 2a3, b3, on ODTS-free substrate the particles average height is 4.5 nm above the surface, on ODTS-coated substrate the value is 2.3 nm above the surface. The height differences verify the NPs are grown inside the pinholes on ODTS-coated substrate.

To study the influence of SAMs growth time to the NPs density. 300 cycles Pd NPs on ODTS SAMs with 0, 10 min and 2 h dip time were characterized with SEM and the statistical analysis results are given in Table S1 of Supplementary Information (SI). As the ODTS growth time increase, both the Pd NPs density and coverage area percentage decrease. The phenomenon could be explained that by increasing the SAMs dip time, the number of defect sites decrease that cause the NPs density decrease too. The observation is similar with the Pt NPs grown on ODTS modified Si substrates with different dip time reported elsewhere^40^. The phenomenon indicates that NPs density could be controlled by varying the ODTS dip time. Meanwhile the standard deviation of particle diameter is 3.8 nm on ODTS-free substrate, 1.9 nm on 10 min ODTS, and 1.7 nm on 2 h ODTS respectively. The decrease of particle diameter's standard deviation may due to the lateral restriction of ODTS SAMs that separate the particles from coalescing thus the size distribution has been narrowed. (Figure S1)

**Figure 3:**
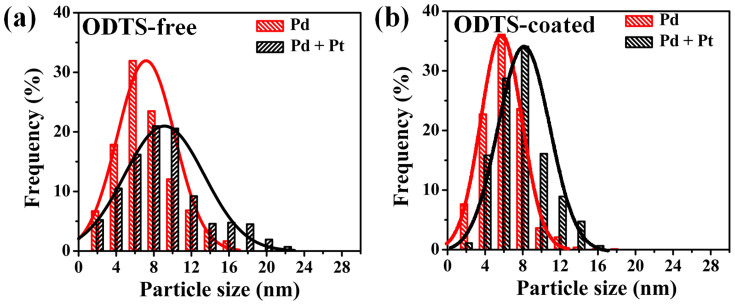
Nanoparticles size distribution on (a) ODTS-free substrate, (b) ODTS-coated substrate. Histograms are made by 1000 particles.

To further elaborate the core shell NPs synthesis process. SEM was performed to obtain the NPs density and size distribution. The images of NPs grown on ODTS-free substrate and ODTS-coated (2 h dip time) substrate are given in Figure S2. On ODTS-free substrate, 300 cycles of Pd NPs' density is 5.6 × 10^3^/um^2^. After 25 cycles Pt ALD process, the particles density increase to 6.3 × 10^3^/um^2^. The phenomenon indicates that new Pt nuclei are formed on substrate during the second stage Pt ALD process. Therefore, the particle density increases. In comparison, on the 2 h ODTS-coated substrate, the NPs density remains constant within measurement error before and after Pt ALD process (2.6 × 10^3^/um^2^ and 2.7 × 10^3^/um^2^, respectively). The unchanged NPs density suggests that the Pt ALD process do not initiate new Pt NPs, exclusively formed core shell Pd/Pt structure^34^. The particle size distributions obtained from SEM images are shown in Figure 3. On ODTS-free substrate (Figure 3a), the particles diameter distribution became broader after Pt ALD process, indicating random growth of Pt on both Si surface and Pd sites, forming a mixture of Pt, Pd, and Pd/Pt NPs. For comparison, on the 2 h ODTS-coated substrate (Figure 3b), the particles size distribution shows a rigid shift ~2 nm in diameter while the shape of histogram curve is similar before and after Pt ALD process. The result gives strong evidence that during Pt ALD process, most Pt was selectively grown on the Pd nuclei due to the ODTS blockage.

To validate the robustness and consistency of AS-ALD process, the same experiments were conducted on ~3 nm ALD Al_2_O_3_ film covered carbon grids to make clearer statistical analysis of particles size and density from TEM top view observation (Figure S3). The particles density changed from 2.6 × 10^3^/um^2^ to 5.4 × 10^3^/um^2^ before and after 25 cycles of Pt deposition. However, on 2 h ODTS SAMs modified substrate, the particles density changed slightly from 1.9 × 10^2^/um^2^ to 1.7 × 10^2^/um^2^ before and after Pt deposition, and no new particles with <3 nm diameter were observed after Pt ALD process (Figure S4) indicating the exclusive formation of Pd/Pt core shell NPs. The same trend observed on both Si wafer and Al_2_O_3_ coated carbon grid suggest that the AS-ALD process could potentially be applied to various substrates.

**Figure 4:**
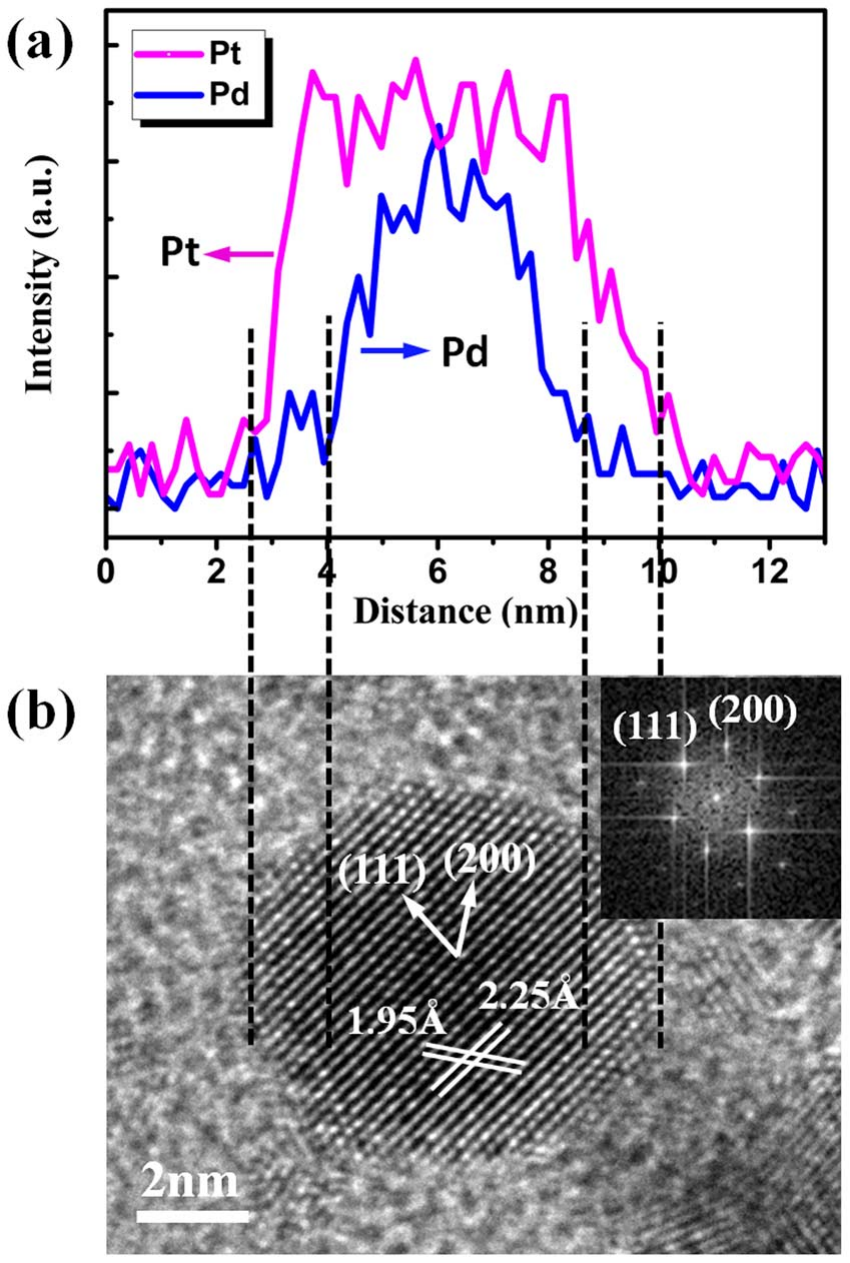
(a) Cross-sectional compositional line profile on a single Pd/Pt core shell NP, (b) bright field HRTEM image of the Pd/Pt core shell NP and corresponding Fourier transform pattern.

The Pd and Pt elemental distribution was analyzed using HAADF-TEM. From EDX line scan in figure 4a, Pd and Pt signals are detected. The compositional line profile shows Pt signal is observed on the surface of the NP. Pd signals are detected only in the inner core of the NP. The results indicate that the NP consists of a Pd core with a diameter of ~4 nm surrounded by a Pt shell with ~1.5 nm thick. Figure 4b shows the high resolution TEM image of a Pd/Pt core shell NP and the corresponding Fourier transform pattern (insert). Since Pt and Pd are very similar in terms of lattice constant and chemical properties, they tend to form core shell NPs with conformal coating. The HRTEM images in bright field confirm that the formed core shell structure NP is of single crystalline nature. The Pt shell lattice parameter for {111} facet is 2.25 Å and for {200} is 1.95 Å. The high resolution TEM image indicates that the Pt shell grown on Pd core exist little lattice fringes. The corresponding Fourier transform pattern further prove that the core shell NP is single crystalline with both {111} and {100} facets enclosed^44^. The selected area electron diffraction (SAED) pattern (Figure S5) indicate the core shell NPs are face centered cubic (fcc) structure with {111} preferred surface orientation. HRTEM and EDX line scan were also performed for the Pd/Pt NPs grown on the 2 h ODTS modified Al_2_O_3_ film deposited on carbon grid. The results are plotted in Figure S6 and indicate that the NP is core shell structure which is consistent with the NP grown on Si wafer.

**Figure 5:**
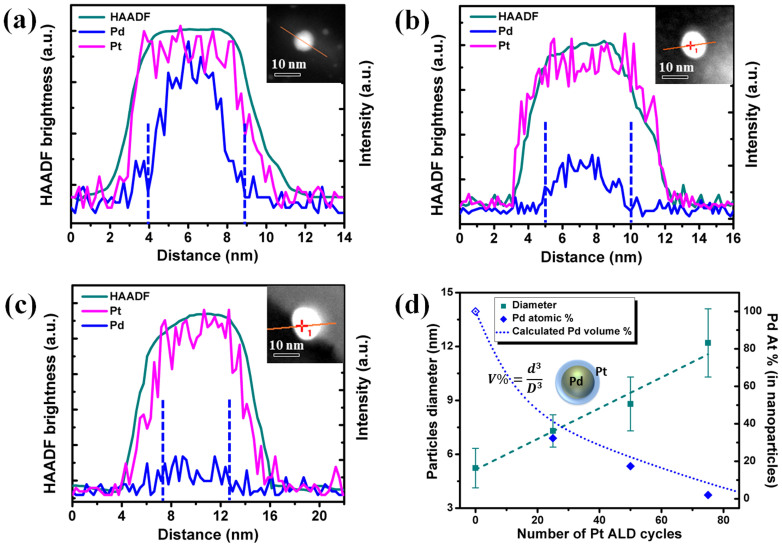
Cross-sectional EDX line profile on a single Pd/Pt core shell NP with (a) 25, (b) 50 and (c) 75 cycles Pt deposited as shell layer. Image (d) represents the core shell NPs' average diameter and Pd atomic percent of the corresponding samples.

Using the developed AS-ALD process, atomically control of shell thickness and bimetallic composition ratio of core shell NPs could be achieved by varying the ALD cycles. Samples with 300 cycles Pd on 2 h ODTS-coated Si substrates were used as initial samples to grow the core shell NPs. To deposit different Pt shell thickness, 25, 50 and 75 Pt ALD cycles in the second stage were performed. Figure 5 displays the STEM-EDX line scan images operated with HAADF mode of the Pd/Pt core shell nanoparticles, the insets represent the corresponding HAADF images of the nanoparticle. Figure 5a, b, c display Pd/Pt core shell NPs with 25, 50 and 75 cycles Pt deposited as shell, respectively. The STEM-HAADF brightness (green line) plotted in Figure 5 stands for the full diameter of the core shell NPs. For figure 5a, b, c, the change of Pt signals are in line with the HADDF brightness indicating that the formation of Pt shell on the surface of the NP. The Pd signal was only detected in the center of the NP and the gap between Pd and Pt signal indicated the shell thickness. The STEM-EDX line scan images demonstrate that the shell thickness increase as Pt ALD cycles increase. The core shell NPs average diameter and atomic percent of Pd/Pt core shell NPs are plotted in Figure 5d. The average diameter of core shell NPs increased near linearly with Pt ALD cycles. The results indicated the Pt shell thickness could be precisely controlled by varying ALD cycles. The shell thickness growth rate is 0.04 nm per cycle from the linear fitting curve of average diameter. The Pd atomic percent decreased as the Pt ALD cycles increased. Assuming the spherical core shell geometry, the Pd volume percent can be calculated through V% = d^3^/D^3^, which d represents the fitted average Pd core diameter, D represents the fitted core shell NPs' full diameter. As Pd and Pt has similar lattice constant, the measured Pd atomic percent is in agreement with the calculated Pd/Pt volume percent.

Then we performed an atmospheric environment anneal to remove SAMs and characterized the structure of Pd/Pt core shell NPs after annealing process. In order to remove the ODTS SAMs from the substrate, the core shell NPs were annealed in atmospheric environment at 400°C for 1 h^45^,^46^. From the spectroscopic ellipsometry measurement for the ODTS SAMs grown on a reference Si wafer, ODTS SAMs was fully removed after the annealing process. The Pd/Pt core shell NPs after such annealing process was characterized with HRTEM and EDX line scan. The results (Figure S7) demonstrate that after the annealing process the lattice constant for Pt surface was unchanged compared with pre-annealing. The EDX line scan profile also indicated that the core shell structure was stable.

**Figure 6:**
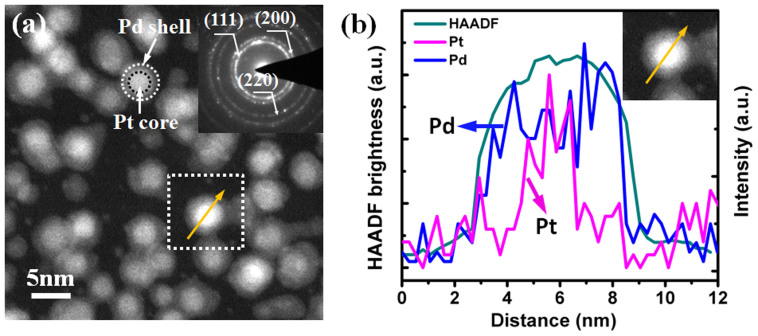
(a) HAADF-TEM image of the Pt/Pd core shell NPs. The sample was oxidized by exposing to gentle N_2_/O_2_ plasma to increase the Z-contrast enabled the core shell structures observation. Inserted image shows the SAED pattern of the sample before oxidizing. (b) TEM-EDX line scanning of the Pt/Pd core shell NP. HAADF image was inserted.

The AS-ALD method is equally flexible in synthesizing inverse Pt/Pd core shell NPs. To fabricate such NPs, we could reverse the order of Pt and Pd ALD deposition sequence. First 100 cycles Pt ALD process was performed to form Pt cores on 2 h ODTS-coated Si substrate. Then deposit Pd shells at 200°C with 150 ALD cycles. To better observe the Pt/Pd core shell structure in HAADF mode, the sample was slightly peroxided by exposing to N_2_/O_2_ plasma for 4 min. Oxidation of the Pd shell would lead to a better Z contrast in the HAADF intensity. Figure 6 shows the Pt/Pd core shell NPs, the lighter brightness present the Pd shell. From the HAADF mode images, all the Pt cores are encapsulated with uniform coated oxidized Pd shells. Pd nucleation on substrate was limited and no new Pd NPs were observed. The core shell structure was also testified using TEM-EDX line scan and demonstrated in figure 6b. The inserted SAED pattern in figure 6a demonstrates the synthesized Pt/Pd core shell NPs (before oxidizing) were fcc structure.

In summary, we demonstrate synthesis of Pd/Pt core shell structure NPs through area selective ALD on unsaturated ODTS SAMs modified substrate. Both core nucleation and shell formation would only take place in the pinholes on ODTS SAMs. The shell layer selectively coated onto the metal cores to form uniform core shell NPs with narrow size distribution. The NPs density could be controlled by varying the ODTS dip time. The size, shell thickness and composition of the NPs can be controlled by varying the ALD cycles. We demonstrate the realization of AS-ALD NPs growth process by fabricating Pd/Pt and Pt/Pd core shell structures, which can be potentially extended to the fabrication of other core shell structures.

## Methods

The substrates were p-type Si (100) wafers with ~2 nm native oxide. All silicon substrates were cleaned ultrasonically by immersing in acetone and followed by anhydrous alcohol. Sufficient de-ionized (DI) water was used to rinse the silicon sample pieces after ultrasonically clean and dried by ultrapure nitrogen blow. The substrates were then exposed to O_2_/N_2_ plasma for 5 min to achieve further surface cleaning and hydrophilic treatment. The SAMs precursor was actadecyltrichlorosilane (C_18_H_37_SiCl_3_, ODTS). The SAMs growing processes were performed in a dry nitrogen atmosphere glovebox without disturbance. Toluene was selected as the solvent with trace water eliminated. The SAMs solution concentration was 10 mmol/L. After SAMs formation, the samples were rinsed sequentially with toluene, acetone and dried by nitrogen flow.

The ALD growth was performed in a commercial Picosun SUNALE^TM^ R200 atomic layer deposition system. Ultrahigh purity nitrogen (99.999%) continuously passed through the chamber and acted as carrier gas. During the whole ALD process the base pressure of the chamber was controlled below 4 Torr. Pd ALD was performed by alternately dosing Pd(II)hexafluoroacetylacetonate (Pd(hfac)_2_, Sigma-Aldrich, 97%) and formalin at 200°C. The Pd(hfac)_2_ was sealed in a stainless steel bottle heated to 50°C. Formalin contains 37% formaldehyde in water with 15% methanol as stabilizer. All the lines are kept at 100°C to avoid condensation. The Pd ALD sequence consisted 1 s pulse of Pd(hfac)_2_ and 2 s pulse of formalin. The 6 s purge of N_2_ was introduced between the precursor pulses to remove the residual precursor. Pt ALD process was performed with (methylcyclopentadienyl)trimethylplatinum (MeCpPtMe_3_) and O_2_ as precursors carried out at 300°C. The Pt source was heated to 65°C to provide enough vapor pressure, the lines was kept at 80°C. The Pt ALD sequence contained 1.6 s pulse of MeCpPtMe_3_ and 2 s pulse of O_2_. The O_2_ pulse pressure was ~1 torr. The 8 s purge of N_2_ was introduced between the precursor pulses to remove the residual precursor.

A field emission scanning electron microscope (FE-SEM, JSM-7600) was employed to image nanoparticles' size and density. ImageJ 1.46 software was utilized to conduct the statistical analysis of the particle diameter and density. The images of nanoparticles were converted into threshold mode to define the NPs and extract the size distribution. An atomic force microscope (Agilent 5500) was employed to measure the morphology of samples' surface and particles' density. The thickness of the ODTS SAMs is measured by spectroscopic ellipsometry (SE, J. A. woollam M2000). Cauchy mode was used to fit the film thickness. The composition and binding energies were examined by X-ray photoelectron spectroscopy (XPS) on an ESCALAB 250 photoelectron spectroscope (Thermo Fisher Scientific Inc.) by using the Al Kα radiation. High angle annular dark field (HAADF) scanning transmission electron microscopy (STEM, FEI Tecnai G2 F30) was performed to characterize the structure of the NPs. For TEM observation, the NPs were scraped off the Si wafer and dispersed on the carbon film grids for HRTEM and EDX line scan characterization.

## Author Contributions

K.C. and R.C. conceived and designed the experiments. K.C. carried out the experiments and wrote the paper. Q.Q.Z. contributed in preparing the self-assembled monolayers. B.S. and R.C. directed the study. All authors participated in discussing and reviewing of the manuscript.

## Supplementary Material

Supplementary Information Supplementary Information
