# Supplementary material for: Controlled Synthesis of Pd/Pt Core Shell Nanoparticles Using Area-selective Atomic Layer Deposition
Source: Sci Rep. 2015 Feb 16;5:8470. doi: 10.1038/srep08470 (PMC4329547; doi:10.1038/srep08470)
Supplement: Supplementary Information [file srep08470-s1.pdf]

Supplementary Information

Controlled Synthesis of Pd/Pt Core Shell Nanoparticles Using Area-selective Atomic Layer Deposition

Kun Cao[1,2], Qianqian Zhu[1,2], Bin Shan[2*], and Rong Chen[1*]

[1]State Key Laboratory of Digital of Manufacturing Equipment and Technology, School of Mechanical Science and Engineering, [2]State Key Laboratory of Material Processing and Die & Mould Technology, School of Materials Science and Engineering, Huazhong University of Science and Technology, 1037 Luoyu Road, Wuhan, Hubei PR China 430074

Corresponding Author
*Email: rongchen@mail.hust.edu.cn, bshan@mail.hust.edu.cn;

Table S1: The statistical analysis of 300 cycles Pd nanoparticles grown on ODTS SAMs modified Si substrate with 0, 10min, 2h dip time.

substrate	Coverage (%)	Density (counts/um^2)	Average diameter (nm)	Diameter σ (nm)
Si	20.8	5.6×10^3	7.1	3.8
10min ODTS	12.1	3.7×10^3	6.3	1.9
2h ODTS	8.7	2.6×10^3	5.1	1.7

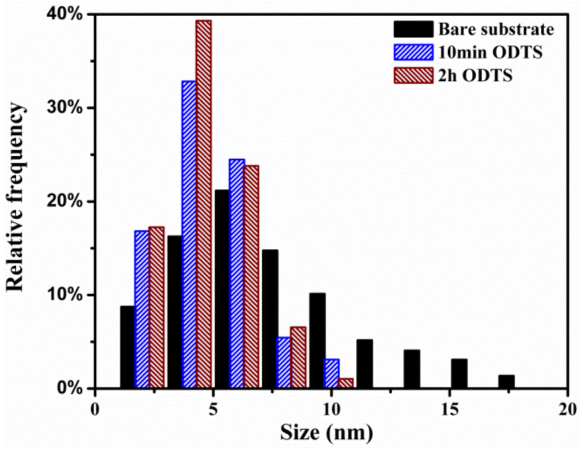

Figure S1. Particles size distribution of 300 cycles Pd on ODTS SAMs modified Si wafer with 0, 10min, 2h dip time.

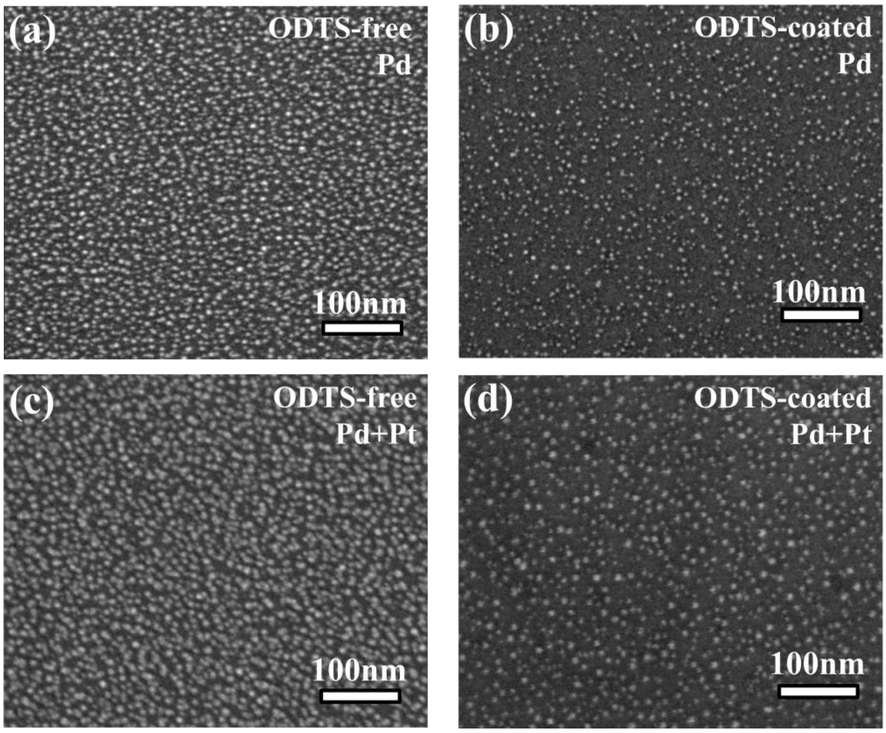

Figure S2. FSEM images of (a) 300 cycles of Pd on ODTS-free Si substrate, (b) 300 cycles of Pd +25 cycles of Pt on ODTS-free Si substrate; (c) 300 cycles of Pd on 2 h ODTS-coated Si substrate, (d) 300 cycles of Pd +25 cycles of Pt on 2 h ODTS-coated Si substrate.

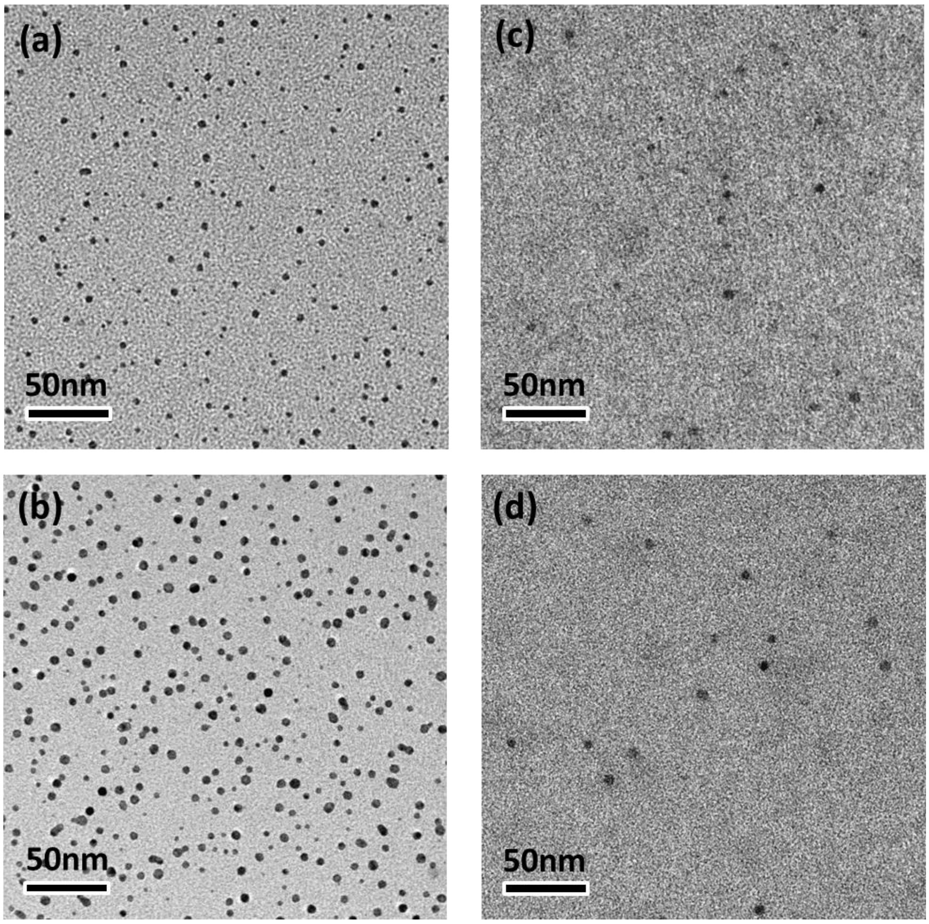

Figure S3. TEM characterization of the noble metal NPs on ~3nm Al_2O_3 pre-covered carbon grids. (a) 300 cycles Pd (b) 300 cycles Pd + 25 cycles Pt on bare Al_2O_3. Images (c) 300 cycles Pd, (d) 300 cycles Pd + 25 cycles Pt demonstrate the NPs grown on the 2h ODTS SAMs modified Al_2O_3 surface.

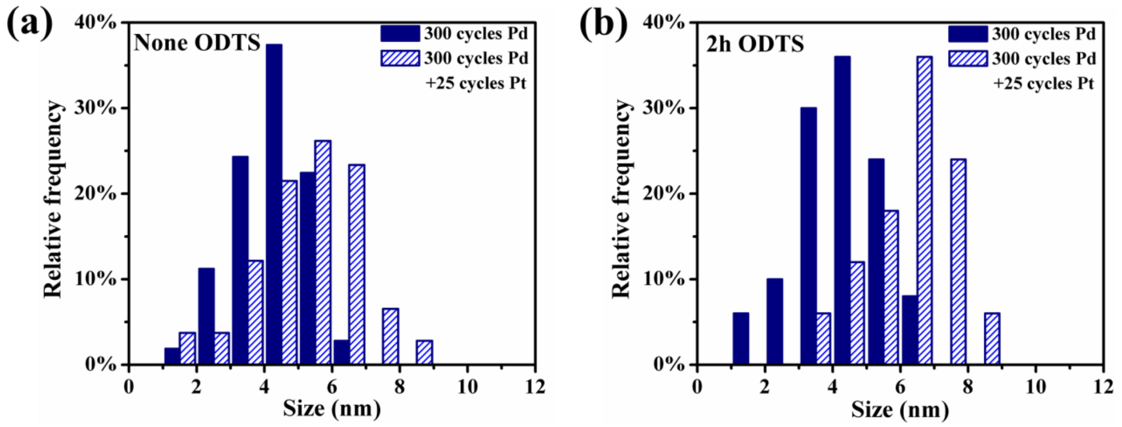

Figure S4. Particles size distribution analysis of the NPs on bare substrate and 2h ODTS modified substrate utilizing the TEM images in Figure S2. Histograms are made by counting 200 particles.

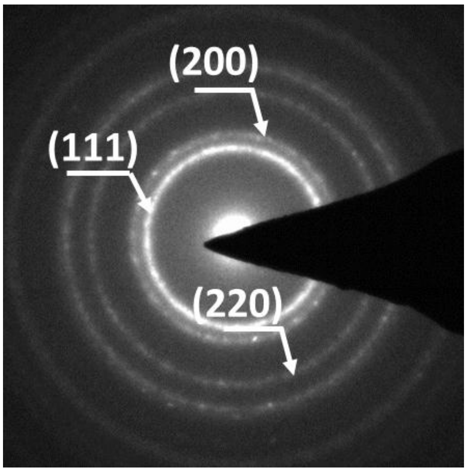

Figure S5. The selected area electron diffraction (SAED) pattern of core shell NPs of 300 cycles Pd followed with 25 cycles Pt on 2h ODTS SAMs.

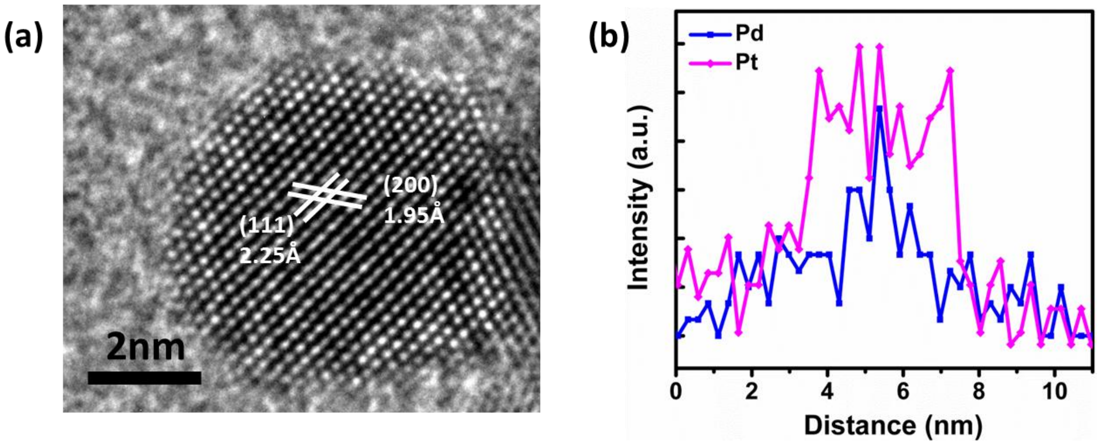

Figure S6. (a) HRTEM images of Pd core (300 cycles) Pt shell (25 cycles) on 2h ODTS modified Al_2O_3 film on carbon grid, (b) corresponding EDX line scan profile.

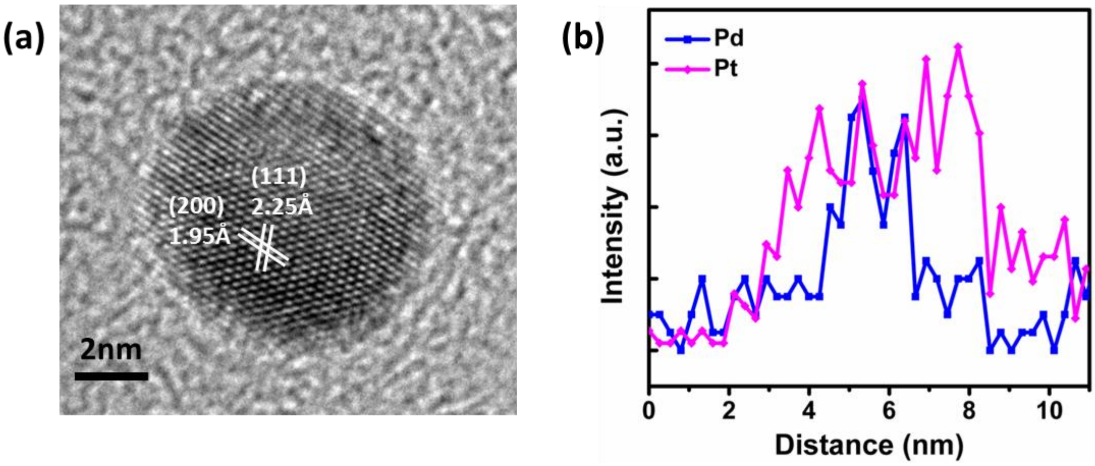

Figure S7. (a) HRTEM images of Pd core (300 cycles) Pt shell (25 cycles) after atmospheric environment annealing at 400 ℃ for 1h, (b) corresponding EDX line scan profile.
